# Advancements in Metacarpal Fracture Management: A Narrative Review of Rehabilitative Strategies

**DOI:** 10.7759/cureus.69970

**Published:** 2024-09-23

**Authors:** Chaitali S Vikhe, Srushti S Daga, Swapnil U Ramteke

**Affiliations:** 1 Department of Sports Physiotherapy, Ravi Nair Physiotherapy College, Datta Meghe Institute of Higher Education and Research, Wardha, IND; 2 Department of Physical Medicine and Rehabilitation, Ravi Nair Physiotherapy College, Datta Meghe Institute of Higher Education and Research, Wardha, IND

**Keywords:** block exercise, hand function, hand injuries, metacarpal fractures, rehabilitation

## Abstract

Metacarpal fractures are prevalent injuries that can significantly impact hand functionality if not managed effectively. This narrative review examines recent advancements in treatment strategies, comparing conservative and surgical interventions, and evaluates the role of early mobilization and innovative rehabilitation techniques. We analyze evidence showing that surgical treatment with low-profile titanium plates provides superior stabilization, enabling earlier mobilization and better functional outcomes compared to conservative methods or K-wire fixation. Early mobilization, facilitated by metacarpal braces or controlled active exercises, enhances recovery and reduces the need for extended physical therapy. We also explore the integration of technology in rehabilitation, which has improved patient adherence and satisfaction. The review highlights the importance of personalized treatment plans and discusses the potential of novel rehabilitation approaches to optimize metacarpal fracture management. Future research should focus on refining these strategies and developing standardized protocols to enhance hand function and patient satisfaction.

## Introduction and background

Metacarpal fractures are a significant portion of hand injuries, representing approximately 35.5% of cases treated in emergency departments. These injuries can result from various causes, including road traffic accidents, falls, and assaults, and can lead to functional impairments if not managed properly. The fifth metacarpal is the most frequently fractured bone in this category, and fractures in the first and fifth metacarpals are often associated with different clinical challenges. Non-border metacarpals are prone to diaphyseal fractures, while border metacarpals typically involve the base or neck [1,2].

The metacarpal bones play a crucial role in maintaining the hand's structure and functionality, contributing to its longitudinal and transverse arches. The articulation of these bones at the carpometacarpal (CMC) joints, particularly the first and fifth CMC joints, makes them susceptible to unstable fractures. The metacarpophalangeal (MP) joints support grip strength and functionality when immobilized in flexion, a common approach in conservative treatment for dorsal apex angulation in shaft or neck fractures [3,4].

Hand fractures are common in the general population, with a higher likelihood among manual laborers and athletes engaged in contact sports like football and boxing. These fractures typically result from direct impact or falls onto the hand, with sports-related activities being a significant contributing factor. Approximately 25% of hand fractures occur during sports activities. Treatment for metacarpal fractures outside of the joint often involves closed reduction and cast immobilization. In cases of intra-articular fractures or unsuccessful closed reduction, internal fixation and open reduction are commonly employed [5,6].

Metacarpal fractures frequently present with swelling and tenderness on the dorsal aspect of the palm, and in some cases, finger deformity is easily noticeable. Among the various hand abnormalities, rotational deformity is the least acceptable. It is crucial to identify rotational deformity in individuals with suspected metacarpal fractures. One of the most obvious signs of rotational malformation is overlapping fingers when presented. Subtle indications of rotational deformity can be revealed by asking the patient to flex their fingers, as they should all point in the direction of the scaphoid tuberosity. When a rotational deformity is present, the fingers will appear to scissors. The rotational deformity can also be assessed using the tenodesis effect, where the fingers flex when the wrist is extended, pointing toward the scaphoid tuberosity. Every nail plate should be parallel to every other plate when viewed at an angle, with a lack of parallelism indicating rotational deformity [7].

Metacarpal fractures are commonly treated by hand surgeons, plastic surgeons, and orthopedic surgeons. These fractures are often observed in young adults who engage in regular physical activities. If left untreated, these fractures can significantly impact the patient's quality of life. Various surgeons manage these fractures differently; even within the same hospital, two doctors may treat the same fracture in different ways. Currently, there are no widely recognized criteria for treatment, with options ranging from conservative maintenance to the use of Kirschner wires (K-wires), plates, and screws [8].

Following type II metacarpal fractures, rehabilitation strategies emphasize a holistic approach to maximize outcomes. The development of clinical hand rehabilitation guidelines focuses on the classification of activities of daily living (ADLs), the appropriate selection of splint types post injury, including grip styles, and the determination of when to initiate early active mobilization. Early occupational and hand therapy is essential for enhancing grip strength and wrist range of motion, particularly when initiated during immobilization. Occupational therapy post-immobilization has also been deemed safe [9]. The review will primarily focus on rehabilitation methods, analyzing their effectiveness in restoring hand function post metacarpal fracture.

## Review

Methodology

Study Selection

This review article conducted a comprehensive search across multiple databases, including PubMed, Google Scholar, Web of Science, and MEDLINE, to evaluate the effectiveness of various physiotherapeutic interventions in the rehabilitation of metacarpal fractures. The search covered the period from 1989 to the present and employed keywords such as "metacarpal fracture," "physiotherapeutic interventions," "rehabilitation strategies," "block exercises," "hand rehabilitation," and "functional improvement." Initially, 97 articles were identified.

Inclusion criteria emphasized studies examining the effects of different rehabilitation approaches on hand functionality and complications. After a thorough review, 24 pertinent papers were selected. These articles addressed the definition and prevalence of metacarpal fractures, diagnostic criteria, implications for daily activities, and treatment modalities, providing significant insights into treatment alternatives and the benefits of physiotherapeutic interventions. The primary objective is to synthesize existing knowledge and highlight effective therapeutic strategies for managing metacarpal fractures.

The review focuses on biomechanical and functional outcomes related to various rehabilitation strategies, with an emphasis on pain control, range of motion (ROM) enhancement, and overall hand function. Findings have important implications for clinical practice, offering evidence-based recommendations for improving post-fracture rehabilitation methods. Further research is needed to address knowledge gaps, particularly concerning long-term outcomes, patient-specific factors, and comparative analyses with alternative rehabilitation methods. This review aims to advance personalized medicine approaches and enhance the quality of care for individuals recovering from metacarpal fractures.

Inclusion and Exclusion Criteria

Inclusion criteria: Studies involving male and female participants within the specified age range with fractures of the second metacarpal, those willing to participate in rehabilitation research, exhibiting pain during finger flexion and extension, and committed to the study.

Exclusion criteria: Studies involving fractures in other hand areas and participants with significantly impaired cognitive functions.

Exclusion Reasons

In this narrative review, the selection of studies followed a structured process to ensure the inclusion of relevant and high-quality data on the rehabilitation of metacarpal fractures. Initially, 97 records were identified through database searches. After removing 43 duplicate records, 54 unique records were screened based on their titles and abstracts. From these, 29 reports were selected for retrieval based on their potential relevance.

Following a detailed full-text review, 14 reports were assessed for eligibility. Nine of these studies met the inclusion criteria and were included in the final review. The remaining records were excluded for the reasons mentioned below.

Articles on diffused hand injuries: Five articles were excluded because they focused on broader hand injuries beyond the specific scope of metacarpal fractures.

Inappropriate study designs: Ten studies were excluded due to methodologies that did not align with the objectives of the review, such as case reports or non-comparative studies.

Irrelevant content: Five articles were excluded for not providing relevant data on rehabilitation methods or outcomes specific to metacarpal fractures.

Insufficient data: Five studies lacked adequate data or results on rehabilitation interventions, making them unsuitable for inclusion.

Additionally, 15 reports could not be retrieved despite extensive efforts. After a full-text review, another five articles were excluded for similar reasons, including a focus on unrelated injuries.

Literature Review

A summary of a review of the literature on management strategies for metacarpal fracture is shown in Table 1.

**Table 1:** Summary of the review of the literature on management strategies for metacarpal fracture. ROM: range of motion; MCP: metacarpophalangeal joints; PRWHE: Patient-Rated Wrist and Hand Evaluation; DASH: Disabilities of the Arm, Shoulder, and Hand.

Serial No.	Authors of publication	Study type/methodology	Study sample	Intervention	Results	Interpretation
1	Rettig et al. [10]	Systematic review	53 athletes	The majority of fractures (46 out of 56) were minimally displaced or undisplaced and managed with simple casting and splinting. Nine of the 10 displaced fractures required open reduction internal fixation with AO-type plates and screws. Three fractures underwent closed reduction with percutaneous pin fixation. Early range-of-motion exercises were initiated.	The mean absence from training or games was 13.7 days, with a longer duration for basketball (19.8 days) compared to football (10.63 days).	The study highlights the effectiveness of various treatment methods for metacarpal fractures in athletes and their impact on recovery time. Personalized treatment plans are important for reducing time away from sports.
2	Statius Muller et al. [11]	Randomized control trial	40 participants with Vth metacarpal fracture	One group received three weeks of therapy with an ulnar gutter plaster cast, followed by mobilization. The second group had a week of treatment with a pressure bandage and delayed mobilization due to discomfort.	35 participants completed the follow-up. Fracture angulation ranged from 15 to 70 degrees with no significant differences between the groups in range of motion, patient satisfaction, pain recovery, or need for physiotherapy.	Pressure bandage followed by mobilization can be effective for boxer's fractures with angulation below 70 degrees. Reducing angulated fractures to less than 70 degrees may not be necessary for preserving the ROM of the fifth MCP joint.
3	Macdonald et al. [12]	Randomized control trial	61 patients	Patients with non-scissoring spiral metacarpal fractures were managed conservatively in a prospective study.	For the non-injured side, the average grip strength was measured at 36.18 kg during the most recent follow-up exam, whereas for the injured side, it was measured at 36.58 kg. After statistical analysis, it was shown that there was no discernible difference in grip strength between the sides that were wounded and those that were not (P = 0.72).	Conservative treatment may be a viable option for managing spiral metacarpal fractures as they do not significantly affect grip strength or functional recovery.
4	Then et al. [13]	Randomized control trial	19 participants	Nineteen people were recruited and randomized into two groups: 10 in the intervention group (gamification) and 10 in the control group (conventional physiotherapy). Assessments of grip strength and composite finger range of motion were made both at the start of the trial and at follow-up appointments. In addition, compliance rates and Patient-Rated Wrist and Hand Evaluation (PRWHE) ratings were recorded.	No significant differences in composite finger range of motion or grip strength were found between the groups. However, the gamification group had better adherence rates (P < 0.05) and PRWHE ratings.	The utilization of gamification through mobile devices offers a cost-effective and secure substitute for conventional hand rehabilitation physiotherapy after metacarpal fractures. This research supports the possibility of creating economical, technology-driven therapeutic treatments in the future.
5	Hirani and Gagal [14]	Randomized control trial	100 participants with metacarpal fracture	A study on 100 individuals with metacarpal fractures compared conservative and surgical treatments at the orthopedics department. Functional outcomes were assessed using the DASH questionnaire, measuring disability on a scale of 0-100. Patient satisfaction was also measured with the Michigan Hand Questionnaire.	The results of the DASH questionnaire showed that a large number of patients scored between 21 and 25, indicating a high level of satisfaction with their treatment. Higher scores on the DASH scale suggest a more severe level of disability. The Michigan Hand Questionnaire also confirmed the high levels of satisfaction among patients regarding their treatment outcomes.	The research highlights the significance of functional results, as patients primarily anticipate the restoration of optimal hand functionality. Physical therapy and mobilization play a critical role in the treatment and rehabilitation of metacarpal fractures.
6	Gülke et al. [15]	Randomized control trial	Sixty individuals diagnosed with metacarpal fractures, excluding the thumb, who received treatment through mobilization-stable open reduction and internal fixation, were part of the study	The participants were randomly prospectively assigned to two groups: one group underwent conventional physical therapy while the other group participated in a home exercise program. Evaluations were carried out at two, six, and 12 weeks after the surgery to assess the results.	Both groups showed improvements in ROM and grip strength by 12 weeks, with 91% and 93% of non-injured hand strength regained.	Both home-based exercise regimens and conventional physical therapy are effective in managing metacarpal fractures, leading to satisfactory functional recovery.
7	Jun et al. [16]	Randomized control trial	The research involved 37 individuals who had been diagnosed with fractures in the proximal phalanges or metacarpals of the second to fifth fingers	Three weeks after the procedure, all patients underwent controlled active exercise after the minimally invasive open reduction and internal fixation. Stress was delivered in the opposite direction of axial loading during this exercise. Three to five weeks following the procedure, the exercise regimen comprised painless active traction in three positions: supination, neutral, and pronation. Range of motion (ROM) in the metacarpophalangeal and interphalangeal joints as well as postoperative radiographs were evaluated.	Significant increases in ROM were noted between six and 12 weeks (P < 0.05). By week 12, 26 patients exceeded 230° ROM in the injured finger.	The study shows that in hand fractures, less extensive dissection of the periosteum and tendons is required when open reduction and internal fixation are performed using minimally invasive procedures. Pain-free traction in supination, neutral, and pronation positions is one of the controlled active exercises that aids in early functional rehabilitation and helps achieve a sufficient range of motion.
8	Küntscher et al. [17]	Prospective study	87 patients with 105 metacarpal fractures were included	Surgical intervention was performed on all fractures. Follow-up information was accessible for 73 individuals (84%) following an average duration of nine months.	Average grip strength compared to the contralateral side was 96% for power grip, 97% for three-finger grip, and 98% for pinch grip in the dominant hand group. The mean DASH score was 6.5 points.	The metacarpal brace used in this study ensured a high degree of patient comfort while providing adequate protection against direct impact. Compared to conventional plaster casts or splints, it did not affect fracture retention. Physical therapy was not as necessary for patients who got early functional treatment with the brace. The results show that the metacarpal brace is a useful tool for early functional treatment after surgery to stabilize metacarpal fractures, which promotes faster healing and less need for extended physical therapy.
9	Randall et al. [18]	Randomized control trial	18 patients with metacarpal fracture	The participants were allocated randomly to one of two groups: the intervention group, which underwent joint mobilization, or the control group, which did not receive any form of treatment.	The treatment group showed a significant increase in ROM compared to the control group (P < 0.05).	Joint mobilization is effective in improving ROM in the metacarpophalangeal joint post-immobilization, supporting its use as a therapeutic intervention for restoring joint function.

Results

A total of nine studies were included in the review, encompassing a range of methodologies such as systematic reviews, randomized controlled trials (RCTs), and prospective studies. The sample sizes in these studies varied from 18 to 100 participants, with most focusing on the interventions for metacarpal fractures, including conservative treatment, surgical options, and rehabilitation methods. The review highlighted key findings: conservative management was effective for non-displaced fractures, with comparable outcomes to surgical approaches in terms of ROM and grip strength. Surgical interventions, particularly open reduction internal fixation (ORIF), were necessary for displaced fractures, and studies emphasized the importance of early mobilization post surgery to prevent stiffness and improve functional recovery. Rehabilitation techniques, such as early active exercises and block exercises, consistently led to improved ROM and grip strength, while novel methods like gamification and home-based exercise programs showed promise in enhancing patient adherence and reducing the dependency on traditional physiotherapy. Additionally, the review noted the potential for digital nerve injuries in metacarpal fractures, especially when displacement occurs, which can significantly impact patient outcomes. Early recognition and treatment of these nerve injuries are critical for optimal recovery.

Discussion

Metacarpal fractures, commonly caused by falls or direct impact, represent a significant portion of hand injuries. Without appropriate treatment, these fractures can severely impair hand function. Over time, a range of treatment strategies for metacarpal fractures has emerged, from conservative approaches to various surgical interventions. This review aims to evaluate the effectiveness of these treatment options and highlight modern rehabilitative strategies that optimize patient outcomes. Complications associated with metacarpal fractures, particularly those involving soft tissue damage, must be addressed regardless of the treatment method chosen. Studies indicate that open wounds and soft tissue injuries increase the risk of poor outcomes, with tendon adhesions and joint stiffness being common complications. These issues are often linked to prolonged immobilization, periosteal stripping, and neurovascular damage. As such, low-profile plates, splinting in functional positions, pain management, and early mobilization are critical components of modern treatment protocols for minimizing complications and promoting recovery [19-21].

Early mobilization is particularly vital in metacarpal fracture rehabilitation. Numerous studies have emphasized its importance in preventing joint stiffness and improving functional outcomes. Advanced rehabilitation techniques, including the use of robotic orthoses, have shown promise in promoting early hand movement and enhancing functional recovery. A study comparing traditional exercises to ADL-based therapy found that the ADL group exhibited greater improvements in grip and pinch strength after a three-week intervention followed by a home-based program. This suggests that incorporating ADL activities into rehabilitation protocols can yield better results than traditional exercises alone [22,23].

Block exercises, which focus on improving ROM and hand function post surgery, are integral to the rehabilitation after metacarpal fractures. These exercises have proven to enhance functional outcomes, even when compared with more invasive procedures such as transverse pinning and intramedullary screw fixation. Compared to standard postoperative physiotherapy, well-structured home exercise programs have also demonstrated significant improvements in hand function and ROM. The management of metacarpal fractures has evolved significantly, particularly in terms of rehabilitation and surgical advancements. The choice of treatment should be personalized, taking into account the patient's age, activity level, and the nature of the fracture [24,25]. Studies have shown that block exercises and mobilization techniques can greatly enhance healing and ROM, particularly for second metacarpal fractures. However, further research, especially randomized controlled trials, is necessary to validate the efficacy of these techniques across a broader range of patient groups. Long-term studies would also help assess the sustainability of treatment outcomes and identify potential complications. Additionally, comparing alternative rehabilitation approaches could provide insight into the most effective treatment modalities for different types of fractures.

Another crucial aspect of metacarpal fracture management is the risk of digital nerve injury, digital nerves run along the metacarpals and are at high risk of injury, either directly from the initial impact or from a displaced fracture compressing or damaging the nerve. Such injuries can significantly affect sensory and motor functions, leading to long-term impairment if not adequately addressed [26]. This review expands on this issue by recognizing the frequency of digital nerve injuries in metacarpal fractures, particularly in displaced cases. The early diagnosis and management of digital nerve injuries are essential to improving patient outcomes and preventing chronic deficits in hand function. Looking ahead, there is a clear need for more research into the physiological and biomechanical mechanisms that underpin these rehabilitation exercises. Emerging technologies like virtual reality (VR) and biofeedback offer promising avenues for enhancing patient engagement and optimizing rehabilitation protocols. Evaluating the cost-effectiveness of such innovative approaches is critical for determining their viability in clinical settings. Closing these research gaps will contribute to developing evidence-based, personalized rehabilitation treatments for metacarpal fractures, improving outcomes across patient populations.

## Conclusions

The literature review emphasizes the significance of personalized treatment strategies for metacarpal fractures. While conservative therapy may still be appropriate in some patients, surgical intervention yields better results in terms of stability, early mobility, and functional recovery particularly when low-profile titanium plates are used. Early mobilization reduces the need for extended physical therapy and significantly improves healing, whether it is achieved by functional bracing or controlled active exercises. Further study ought to focus on optimizing rehabilitation procedures and exploring the benefits of novel approaches such as gamification, block exercises, hand rehabilitation, and mobilization with mobility in enhancing patient outcomes and adherence.
